# *Quercus infectoria* Gall Ethanolic Extract Accelerates Wound Healing through Attenuating Inflammation and Oxidative Injuries in Skin Fibroblasts

**DOI:** 10.3390/antiox13091094

**Published:** 2024-09-09

**Authors:** Suttiwan Wunnoo, Decha Sermwittayawong, Rachanida Praparatana, Supayang Piyawan Voravuthikunchai, Chanawee Jakkawanpitak

**Affiliations:** 1 Center of Antimicrobial Biomaterial Innovation-Southeast Asia, Faculty of Science, Prince of Songkla University, Hat Yai, Songkhla 90110, Thailand; suttiwan.w@psu.ac.th (S.W.); supayang.v@psu.ac.th (S.P.V.); 2 Center of Excellence for Biochemistry, Faculty of Science, Prince of Songkla University, Hat Yai, Songkhla 90110, Thailand; decha.s@psu.ac.th; 3 Division of Health and Applied Sciences, Faculty of Science, Prince of Songkla University, Hat Yai, Songkhla 90110, Thailand; 4 Faculty of Medical Technology, Prince of Songkla University, Hat Yai, Songkhla 90110, Thailand; rachanida.p@psu.ac.th

**Keywords:** antioxidant activity, hydrogen peroxide, inflammation, oxidative stress, *Quercus infectoria* gall, wound healing

## Abstract

*Quercus infectoria* Olivier (Fagaceae) nutgall, a traditional Asian medicine, is renowned for its efficacy in treating wounds and skin disorders. Although the gall extract has shown promising results in accelerating wound healing in diabetic animal models, its mechanisms, particularly the effects on redox balance, remain poorly understood. This study aims to investigate the effects and mechanisms of *Q. infectoria* gall ethanolic extract (QIG) on wound healing in fibroblasts, with a specific emphasis on its modulation of oxidative stress. Hydrogen peroxide (H_2_O_2_)-treated L929 cells were used as an in vitro model of oxidation-damaged fibroblasts. QIG exhibited potent antioxidant activity with 2,2-diphenyl-1-picrylhydrazyl (DPPH), 2,2′-azino-bis(3-ethylbenzothiazoline-6-sulfonic acid (ABTS), and ferric reducing antioxidant power (FRAP) assay values of 305.43 ± 7.48, 508.94 ± 15.12, and 442.08 ± 9.41 µM Trolox equivalents (TE)/µg, respectively. Elevated H_2_O_2_ levels significantly reduced L929 cell viability, with a 50% lethal concentration of 1.03 mM. QIG mitigated H_2_O_2_-induced cytotoxicity in a dose-dependent manner, showing protective effects in pre-, post-, and co-treatment scenarios. QIG significantly reduced H_2_O_2_-induced intracellular reactive oxygen species production and inflammation-related gene expression (*p* < 0.05). Additionally, at 25 µg/mL, QIG remarkably improved wound closure in H_2_O_2_-treated L929 cells by approximately 9.4 times compared with the H_2_O_2_ treatment alone (*p* < 0.05). These findings suggest QIG has potential therapeutic applications in wound healing, mediated through the regulation of oxidative stress and inflammatory response.

## 1. Introduction

Oxidative stress arises from an imbalance between the generation of reactive oxygen species (ROS) and the body’s antioxidant defenses. Elevated ROS levels are recognized as a contributing factor in chronic wounds, especially in diabetes mellitus [1]. Persistent hyperglycemia in diabetics promotes ROS production, including hydrogen peroxide (H_2_O_2_), through mechanisms such as glucose autoxidation and enzymatic reactions [2]. Excessive H_2_O_2_ production leads to oxidative damage that exacerbates inflammation [3], induces apoptosis [4], and disrupts the function of cells like keratinocytes and fibroblasts, thereby delaying the healing process [5]. The detrimental effects of oxidative stress on wound healing are multifaceted. ROS, particularly H_2_O_2_, can directly damage cellular components, including lipids, proteins, and DNA [6]. This damage triggers a cascade of harmful cellular responses, including the activation of pro-inflammatory signaling pathways (e.g., nuclear factor kappa light chain enhancer of activated B cells (NF-κB)) [7]. As a result, chronic inflammation hinders the progression of the normal wound healing process by impairing the remodeling phase, leading to prolonged tissue injury and scarring [8]. Therefore, addressing H_2_O_2_-mediated oxidative stress has emerged as a promising therapeutic strategy for improving wound healing. This approach may involve using antioxidants or targeted therapies to mitigate ROS levels and restore redox balance in the wound microenvironment.

In fibroblasts, oxidative stress interferes with their critical roles in wound healing, including extracellular matrix (ECM) synthesis, tissue remodeling, and wound contraction [9]. H_2_O_2_-induced oxidative stress can alter the expression of genes involved in ECM production, resulting in reduced collagen synthesis and impaired ECM integrity [10]. This disruption compromises the structural framework necessary for effective tissue repair. Additionally, oxidative stress can induce senescence in fibroblasts, a state in which cells lose their proliferative capacity and fail to migrate effectively to the wound site [9,11]. Senescent fibroblasts secrete pro-inflammatory cytokines and matrix-degrading enzymes, exacerbating tissue damage and inhibiting proper wound closure [12]. Moreover, oxidative stress promotes fibroblast apoptosis through the activation of mitochondrial pathways and the disruption of redox-sensitive signaling cascades [13]. This reduction in viable fibroblast numbers directly impacts wound healing by diminishing the cellular workforce required for tissue regeneration.

*Quercus infectoria* gall, a natural product derived from the oak tree *Q. infectoria* Olivier, has a long history in traditional medicine, particularly in regions of Asia and the Middle East. Numerous studies have extensively investigated the therapeutic potential of *Q. infectoria* gall to validate their traditional uses such as antimicrobial, antitumor, anti-inflammatory, and antioxidant activities [14,15,16]. In Asian countries, the nutgalls have been utilized to treat various diseases, including enteritis, dysentery, halitosis, intestinal dysmotility, tympanitis, pharyngolaryngitis, alopecia areata, and dental caries [17]. In Thailand, *Q. infectoria* galls have been traditionally used for wound therapy for many years. They are an important ingredient in traditional Thai recipes for wound treatment, addressing conditions such as aphthous ulcers and diabetic wounds [18,19]. Previous studies demonstrated that *Q. infectoria* gall hydroethanolic extract and formulations containing *Q. infectoria* extract can accelerate wound healing in diabetic mouse and rat models [20,21]. Additionally, *Q. infectoria* ethanolic extract has been used to treat diabetic patients with chronic ulcers, where rapid wound healing was observed in the study group compared with the standard wound treatment with normal saline solution [18]. Although the impact of the nutgalls on diabetic wound healing is well documented, the effects of *Q. infectoria* gall extract on H_2_O_2_-mediated oxidative stress, a key factor in diabetic wounds, remain poorly understood.

Several bioactive metabolites have been identified in *Q. infectoria* gall extract, including phenolic acids, flavonoids, triterpenoids, and steroids [17]. Among these, tannic acid is the most abundant, comprising 50–70% of the nutgalls [22]. A recent study showed that tannic acid can alleviate inflammation by reducing myeloperoxidase enzyme activity in a paw edema model [23]. In addition, tannic acid exhibits gastroprotective effects by counteracting oxidative stress and reducing pro-inflammatory cytokines like TNF-α, IL-1β, and IL-6, while increasing the anti-inflammatory cytokine IL-10 [24]. Based on these properties, we expect that the gall extract may protect fibroblasts from oxidative damage and prolonged inflammation, thereby promoting wound healing in diabetic conditions. This study aims to investigate the effects of *Q. infectoria* gall ethanolic extract on L929 skin fibroblasts under an H_2_O_2_-induced oxidative stress model. We conducted a series of experiments to determine whether the gall extract can reduce H_2_O_2_-induced cytotoxicity, decrease intracellular ROS levels, and mitigate inflammation in L929 cells. In addition, the effects of H_2_O_2_ and *Q. infectoria* gall extract on fibroblast migration, a critical step for skin wound healing, were analyzed and discussed in this study.

## 2. Materials and Methods

### 2.1. Plant Material and Reagents

The galls of *Q. infectoria* were purchased from a local market and authenticated, with a voucher specimen (No. SKP225170901) deposited at the Herbarium of the Faculty of Pharmaceutical Science, Prince of Songkla University, Hat Yai, Songkhla, Thailand. For cell culture, Dulbecco’s Modified Eagle Medium (DMEM), fetal bovine serum (FBS), Antibiotic–Antimycotic, phosphate-buffered saline (PBS), and trypsin-EDTA were purchased from Gibco, Thermo Fisher Scientific (Waltham, MA, USA). For chemical analyses, hydrogen peroxide, 2,2-diphenyl-1-picrylhydrazyl (DPPH), 2,2′-Azino-bis(3-ethylbenzothiazoline-6-sulfonic acid) diammonium salt (ABTS), 3-[4,5-dimethylthiazol-2-yl]-2,5 diphenyl tetrazolium bromide (MTT), ferric chloride hexahydrate, 2′,7′-dichlorodihydrofluorescein diacetate (DCFH-DA), and analytical standards including tannic acid, gallic acid, and L-ascorbic acid were purchased from Sigma-Aldrich (St. Louis, MO, USA). All other reagents were of analytical grade and were purchased from Krungthepchemi Co., Ltd. (Bangkok, Thailand).

### 2.2. Preparation of Q. infectoria Gall Ethanolic Extract

The gall extract was prepared following the method described by [25]. Briefly, 100 g of gall powder was mixed with 500 mL of 95% ethanol at room temperature for 7 days. After filtration, the excess ethanol was removed using a rotary evaporator at 60 °C. The crude extract of *Q. infectoria* gall was stored at room temperature and protected from light. The extract solution was prepared by dissolving the crude extract in distilled water to a final concentration of 50 mg/mL, followed by sterilization using a syringe filter (0.22 µm pore size) before use.

### 2.3. High-Performance Liquid Chromatography (HPLC) Analysis of Q. infectoria Gall Extract

The determination of tannic acid from *Q. infectoria* gall extract was carried out using high-performance liquid chromatography. Briefly, 300 µg of the sample was dissolved in 1 mL of water. The solution was then filtered using a 0.2 µm nylon filter membrane and injected into an HPLC system (Agilent 1260 Infinity). The separation was achieved on an ACE 5 C18 (4.6 × 150 mm) column. The mobile phase consisted of acetonitrile and 0.1% phosphoric acid in water (5:95, *v*/*v*). The flow rate was set to 1 mL/min with an injection volume of 20 µL. The column temperature was maintained at 25 °C. The sample peaks were identified by comparison with a standard tannic acid solution at 280 nm.

### 2.4. Evaluation of Antioxidant Activity

To compare the antioxidant capacities of *Q. infectoria* gall extract with those of compounds (tannic acid, gallic acid, and L-ascorbic acid), DPPH, ABTS, and FRAP assays were conducted following previously described methods [26].

#### 2.4.1. DPPH Radical Scavenging Assay

The DPPH assay was performed by combining 20 µL of the samples with 180 µL of 0.2 mM DPPH solution. The mixture was incubated for 40 min at room temperature in the dark, and then the absorbance at 517 nm was measured. A Trolox solution with concentrations ranging from 100 to 700 µM was used to generate a standard curve. The assay was performed in triplicate and the results were expressed as µM of Trolox equivalents (TE) per 1 µg of the samples.

#### 2.4.2. ABTS Radical Scavenging Assay

First, the ABTS·^+^ working reagent (14 mM ABTS solution and 4.9 mM potassium persulfate in a ratio of 1:1) was prepared and adjusted to 0.700 ± 0.025 at 734 nm using distilled water. Then, 20 µL of the samples was mixed with 180 µL of the ABTS·^+^ working solution and incubated in the dark for 15 min at room temperature. The absorbance at 734 nm was measured. The standard curve was prepared using a Trolox solution with concentrations ranging from 100 to 500 µM. The assay was performed in triplicate, and the results were expressed as µM of Trolox equivalents (TE) per 1 µg of the sample.

#### 2.4.3. Ferric Reducing Antioxidant Power (FRAP) Assay

The assay was conducted by mixing 20 µL of the samples with 180 µL of pre-warmed (37 °C) working FRAP solution (300 mM acetate buffer pH 3.6, 2,4,6-tripyridyl-s-triazine (TPTZ) in 40 mM hydrochloric acid, and 20 mM ferric chloride hexahydrate in a ratio of 10:1:1, respectively). The mixtures were incubated in the dark for 30 min at room temperature, followed by measurement of the absorbance at 593 nm. The standard curve was created using Trolox ranging from 100 to 700 µM. The assay was performed in triplicate and the results were expressed as µM of Trolox equivalents (TE) per 1 µg of the samples.

### 2.5. Cell Culture Condition

The L929 murine fibroblast cell line (ATCC) was cultured in complete Dulbecco’s modified Eagle’s medium (cDMEM) containing DMEM high-glucose medium, L-glutamine, sodium pyruvate, 10% fetal bovine serum (FBS), and 1% Antibiotic–Antimycotic. The cells were incubated at 37 °C in a humidified atmosphere with 5% CO_2_.

### 2.6. H_2_O_2_ Dose Optimization and Cell Viability Assay

To evaluate the cytotoxic effects of H_2_O_2_ on L929 fibroblasts, cells were seeded into a 96-well plate at a density of 3 × 10^4^ cells/well and cultured for 16–18 h. Cells were then exposed to various concentrations of H_2_O_2_ ranging from 0.25 to 8 mM for 24 h. Cell viability was determined using a 3-[4,5-dimethylthiazol-2-yl]-2,5 diphenyl tetrazolium bromide (MTT) assay. Briefly, cells were incubated with 0.5 mg/mL MTT in cDMEM for 4 h. After removing the supernatant, 200 µL of DMSO was added to dissolve the formazan crystals. Absorbance was measured at 570 nm using a microplate reader, Tecan Spark (Männedorf, Switzerland). The percentage of cell viability was calculated using the following formula: (OD value of sample)/(OD value of control) × 100. The LC_50_ (50% lethal concentration) of H_2_O_2_ for L929 cells was determined based on the cell viability results and was applied in all subsequent experiments.

To investigate the effect of *Q. infectoria* gall extract on H_2_O_2_-induced injury in L929 fibroblasts, L929 cells were seeded into a 96-well plate and cultured similarly as described above. The cells were then subjected to the following treatments: (1) the extract at concentrations ranging from 12.5 to 200 µg/mL for 24 h; (2) co-treatment with H_2_O_2_ (1 mM) and different doses of the gall extract (12.5 to 100 µg/mL) for 24 h; (3) 25 µg/mL of either tannic acid, gallic acid, L-ascorbic acid, or *Q. infectoria* gall extract in the presence or absence of H_2_O_2_ (1 mM) for 24 h; and (4) the gall extract (25 µg/mL) and/or H_2_O_2_ (1 mM) at different time intervals, as depicted in Section 3.7. After treatment, the cell morphology was observed, and the images were captured using a microscope at an objective of ×20, CKX53, Olympus (Tokyo, Japan). Cell viability was determined using the MTT assay, with results expressed as a percentage of the control.

### 2.7. Detection of Intracellular ROS Levels

Total intracellular ROS were determined using 2′,7′-dichlorodihydrofluorescein diacetate (DCFH-DA) staining. In brief, L929 fibroblasts were seeded into a 96-well plate at a density of 3 × 10^4^ cells/well and cultured at 37 °C with 5% CO_2_ for 16–18 h. The cells were treated with 1 mM H_2_O_2_ in the presence or absence of different doses of *Q. infectoria* gall extract (12.5 and 25 µg/mL) for 24 h. Subsequently, the cells were incubated with cDMEM medium containing 10 µM DCF-DA for 30 min at 37 °C, followed by washing twice with 1× PBS. Images of intracellular ROS formation were captured under an inverted fluorescence microscope at an objective of ×20, CKX53, Olympus (Tokyo, Japan). To quantify ROS levels, the cells were lysed using lysis buffer (0.05% Triton X-100 in 1× PBS). The fluorescence intensity was determined at excitation and emission wavelengths of 485 and 528 nm, respectively, using a microplate reader, Tecan Spark (Männedorf, Switzerland). The results were expressed as fold changes relative to the control group.

### 2.8. Scratch Wound Healing Assay

The wound-healing potential of *Q. infectoria* gall extract under H_2_O_2_-induced oxidative stress in fibroblasts was evaluated by monitoring the level of cell migration. L929 cells were seeded into a 24-well plate at a density of 1 × 10^5^ cells/well and incubated at 37 °C in a humidified atmosphere containing 5% CO_2_ for 2 days. Subsequently, the cell layer was scratched using a 200 µL sterile tip and washed twice with 1× PBS. Then, cells were subjected to the following conditions: (1) H_2_O_2_ at concentrations ranging from 0.125 to 1 mM and (2) 1 mM H_2_O_2_ with or without different doses of *Q. infectoria* gall extract at 12.5 and 25 µg/mL. After treatment, images of the scratched area were captured at 0, 24, and 48 h. The percentage of wound closure was analyzed using ImageJ software version 1.54f (National Institutes of Health, Bethesda, MD, USA) and calculated using the following equation:

Wound Closure (%) = [(A_0_ − A_t_)/A_0_] × 100

where A_0_ and A_t_ are the area of the wound at day 0 and the area of the wound at the indicated time, respectively.

### 2.9. Quantitative Reverse Transcription PCR (RT-qPCR)

Total RNA extraction and complementary DNA (cDNA) synthesis were carried out following the protocol described in a previous study [27]. Briefly, the first-strand cDNA was generated from 1 µg of total RNA, and real-time PCR analysis was conducted using qPCRBIO SyGreen Blue Mix (PCR Biosystems, London, UK) with a real-time PCR machine (Stratagene Mx3005P, Agilent Technologies, Santa Clara, CA, USA). The primer sequences used in this study are presented in Table 1. The assays were run for 40 cycles at an annealing temperature of 60 °C. Data analysis was performed using the 2^−ΔΔCt^ method. Relative gene expression was normalized against the reference gene (*Actb*) and compared with the untreated control group.

### 2.10. Statistical Analysis

All experiments were performed in triplicate and independently repeated at least three times. Data are presented as the mean ± SD. Statistical analyses were performed using SPSS software (version 27.0; IBM Corp., Armonk, NY, USA). One-way analysis of variance, followed by Duncan’s multiple-range test, was used to analyze the significance of differences between groups. Different letters between two datasets indicate statistically significant differences (*p* < 0.05).

## 3. Results and Discussion

### 3.1. HPLC Determination of Tannic Acid

**Figure 1:**
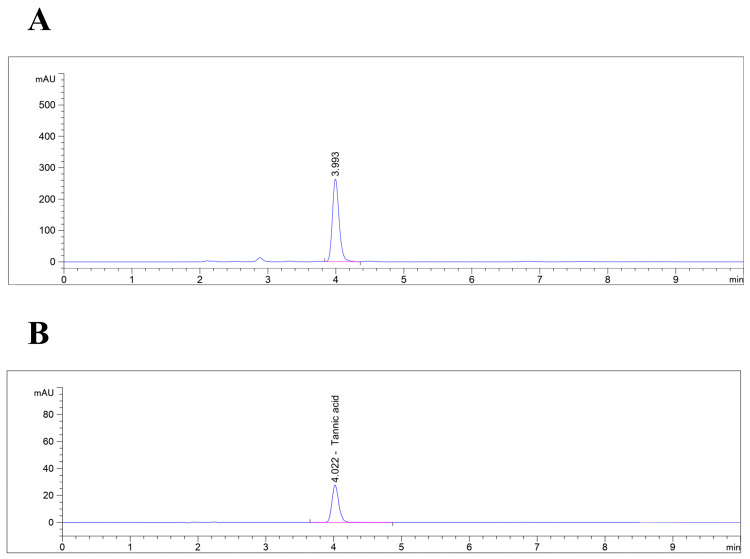
HPLC chromatogram of (**A**) *Q. infectoria* gall extract and (**B**) standard tannic acid. The clear peak at retention time ~4 min indicated the presence of tannic acid.

The phytochemical profile of *Q. infectoria* is well characterized, with studies showing that the extract of *Q. infectoria* galls contains a high amount of tannic acid (50–70%) along with some phenolic acids, such as gallic, ellagic, and syringic acids [17,22,28]. Due to this high concentration, tannic acid can be used as a marker to identify the nutgall extract. In this study, the HPLC method was optimized to detect tannic acid, resulting in a distinct peak with minimal interference from other compounds. The HPLC analysis of *Q. infectoria* gall extract successfully identified tannic acid as a major component. The chromatogram of the extract displayed a prominent peak at a retention time of approximately 3.993 min (Figure 1A), which closely matched the retention time of 4.022 min observed for the standard tannic acid (Figure 1B). This close alignment in retention times confirms the presence of tannic acid in the gall extract. Additionally, a strong tannic acid peak found in the HPLC analysis was consistent with a previous study by Iylia et al. [22], suggesting that the high tannic acid content is a characteristic feature of *Q. infectoria* gall extract. This finding not only validates the use of tannic acid as a marker for identifying *Q. infectoria* extract but also supports the reproducibility of the HPLC method for detecting this compound. The consistency between the current analysis and previous study suggests that the high concentration of tannic acid in *Q. infectoria galls* is a reliable indicator of its phytochemical profile.

### 3.2. In Vitro Antioxidant Activity

To evaluate the antioxidant capacities of *Q. infectoria* gall extract, DPPH, ABTS, and FRAP assays were conducted. Standard tannic acid, gallic acid, and L-ascorbic acid were used as positive controls. In this study, antioxidant values were expressed in µM Trolox equivalents (TE) per µg of sample to ensure consistency with the evaluation of *Q. infectoria* gall extract, which was measured on a weight basis. This approach allows for a direct comparison between the antioxidant activities of the extract and the individual compounds. As shown in Table 2, gallic acid exhibited the strongest antioxidant activity, followed by tannic acid and L-ascorbic acid when compared by the same weight. The antioxidant activities of *Q. infectoria* gall extract were found to be comparable to those of tannic acid. Additionally, the extract exhibited higher antioxidant capacity than L-ascorbic acid, with DPPH, ABTS, and FRAP assay values of 305.43 ± 7.48 vs. 153.02 ± 11.81, 508.94 ± 15.12 vs. 223.70 ± 18.78, and 442.08 ± 9.41 vs. 131.73 ± 22.98 µM TE/µg (*p* < 0.05), respectively. The results indicate that *Q. infectoria* gall extract is a potent source of antioxidants, with these effects likely attributed to tannic acid, the main constituent of the extract. Tannic acid is well known for its antioxidant properties, particularly in neutralizing free radicals [29]. This ability to scavenge free radicals plays a crucial role in preventing oxidative stress, which is implicated in various chronic diseases and aging processes. Moreover, tannic acid has been shown to protect cellular components from oxidative damage by donating electrons to unstable free radicals, thereby stabilizing them and preventing further cellular injury [30]. This potent antioxidant activity also contributes to the effectiveness of tannic acid in reducing inflammation and promoting wound healing, as oxidative stress is a key factor in both processes. Therefore, it is suggested that *Q. infectoria* gall extract may serve as a valuable therapeutic agent in preventing oxidative stress-related cellular damage and supporting wound healing.

### 3.3. Determination of H_2_O_2_ and Quercus infectoria Gall Extract Treatment Model for L929 Fibroblasts

**Figure 2:**
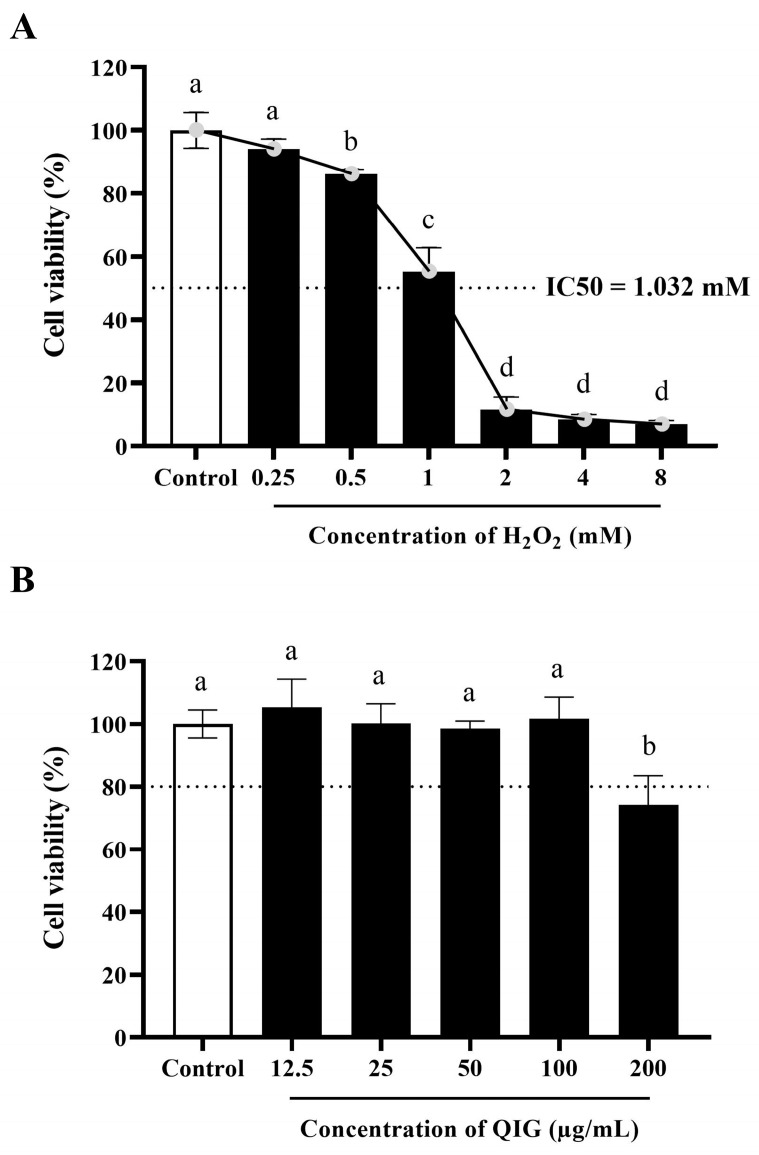
The effects of H_2_O_2_ and *Quercus infectoria* gall extract (QIG) on cell viability of L929 fibroblasts. (**A**) Cells were treated with different doses of H_2_O_2_ ranging from 0.25 to 8 mM for 24 h. (**B**) Cells were exposed to various concentrations of QIG from 12.5 to 200 µg/mL for 24 h. The viability of cells was determined using an MTT assay. The values are expressed as relative to the control group (untreated cells). Values are representative of three independent experiments performed in triplicate and are expressed as mean ± SD (error bars). Different letters indicate statistically significant differences (*p* < 0.05).

H_2_O_2_ has been extensively studied for its cytotoxic effects, with different cell types exhibiting varying sensitivity to H_2_O_2_. For instance, the cell viability of human neuroblastoma cells (SH-SY5Y), human umbilical vein endothelial cells (HUVECs), and murine RAW 264.7 macrophages was decreased by 50% when exposed to H_2_O_2_ at concentrations of 60 µM, 300 µM, and 1.25 mM, respectively [31,32,33]. Thus, to investigate the cytotoxic effect of H_2_O_2_ on L929 fibroblasts, various concentrations of H_2_O_2_ ranging from 0.25 to 8 mM were tested. The results demonstrated that H_2_O_2_ decreased the cell viability of L929 cells in a dose-dependent manner (Figure 2A). The initial concentration of H_2_O_2_ that significantly reduced L929 cell viability was 0.5 mM (cell viability of 86.24 ± 1.4%) (*p* < 0.05), and the lethal concentration 50% (LC_50_) of H_2_O_2_ for L929 cells was 1.032 mM. This value is consistent with the finding from a previous study on human fibroblasts (hFB), where an LC_50_ of 1 mM was reported [5]. As a result, H_2_O_2_ at 1 mM was selected and applied in all subsequent experiments.

To evaluate the cytotoxicity of *Q. infectoria* gall extract on L929 cells, gall extract concentrations ranging from 12.5 to 200 µg/mL were tested. Cell viability exceeding 80% was considered non-toxic. As depicted in Figure 2B, no cytotoxic effects were observed in cells treated with the extract at concentrations up to 100 µg/mL. Previous studies have shown that the effects of *Q. infectoria* gall on cell viability are dose-dependent. For example, at high concentrations (>300 µg/mL), gall extract has been demonstrated to trigger autophagic cell death in colorectal cancer cells [34], while at low concentrations (~10 µg/mL), *Q. infectoria* gall extract was found to promote proliferation in human fetal osteoblast cell lines [35]. However, in this study, the gall extract exhibited minimal effects on L929 fibroblast proliferation, indicating potential cell-type-specific responses.

### 3.4. Quercus infectoria Gall Extract Potentially Protects H_2_O_2_-Induced Cytotoxicity in L929 Fibroblasts

**Figure 3:**
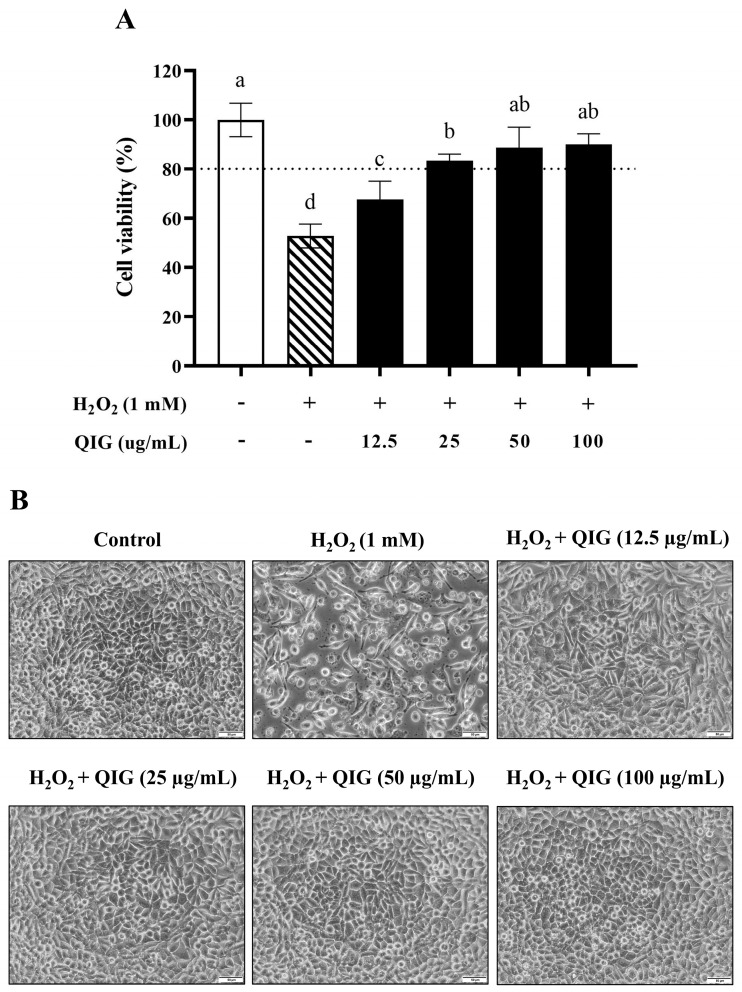
The protective effects of *Quercus infectoria* gall extract (QIG) against H_2_O_2_-induced injury in L929 fibroblasts. Cells were treated with 1 mM of H_2_O_2_ in the presence or absence of QIG ranging from 12.5 to 100 µg/mL for 24 h. (**A**) Cell viability was assessed using an MTT assay. The values are expressed as relative to the control group (untreated cells). Values are representative of three independent experiments performed in triplicate and are expressed as mean ± SD (error bars). Different letters indicate statistically significant differences (*p* < 0.05). (**B**) Representative microscopic images at a magnification of 200×. Scale bar = 50 µm.

To investigate the influence of *Q. infectoria* gall extract on H_2_O_2_-induced cytotoxicity, L929 cells were treated with H_2_O_2_ in the presence or absence of various concentrations of the gall extract (12.5, 25, 50, and 100 µg/mL). Our findings revealed that treatment with 1 mM H_2_O_2_ significantly reduced the cell viability of L929 fibroblasts to 52.80 ± 4.87%, and the morphology of the cells was noticeably altered, with most cells displaying shrinkage and loss of cell-to-cell contact. Interestingly, the presence of *Q. infectoria* gall extract significantly increased cell viability in a dose-dependent manner compared to the cells treated with H_2_O_2_ alone (*p* < 0.05) (Figure 3A). In addition, the extract at concentrations of 25 to 100 µg/mL effectively attenuated H_2_O_2_-induced morphological changes in L929 cells (Figure 3B). These results suggest that *Q. infectoria* gall extract can protect L929 fibroblasts against H_2_O_2_-induced cytotoxicity.

H_2_O_2_ is known to induce various cellular effects, with apoptosis being a common outcome. Numerous studies have demonstrated that H_2_O_2_ can induce apoptosis in multiple cell types, including human umbilical vein endothelial cells (HUVECs), human neuroblastoma cells (SH-SY5Y), and human fibroblasts (hFBs) [5,32,33]. In L929 fibroblasts, H_2_O_2_ has been reported to trigger the mitochondria-mediated pathway, the mitogen-activated protein kinase (MAPK) pathway, and caspase 8/9 activity, resulting in apoptosis activation [36]. The results in Figure 3B revealed that the morphology of L929 fibroblasts dramatically changed after H_2_O_2_ exposure. Most cells exhibited shrinkage and reduced size, which are the morphological hallmarks of apoptotic cells [37]. Collectively, *Q. infectoria* gall extract may protect L929 fibroblasts against H_2_O_2_-induced cytotoxicity by inhibiting apoptotic progression.

### 3.5. Quercus infectoria Gall Extract Ameliorates Intracellular ROS Generation upon H_2_O_2_-Induced Oxidative Stress in L929 Fibroblasts

**Figure 4:**
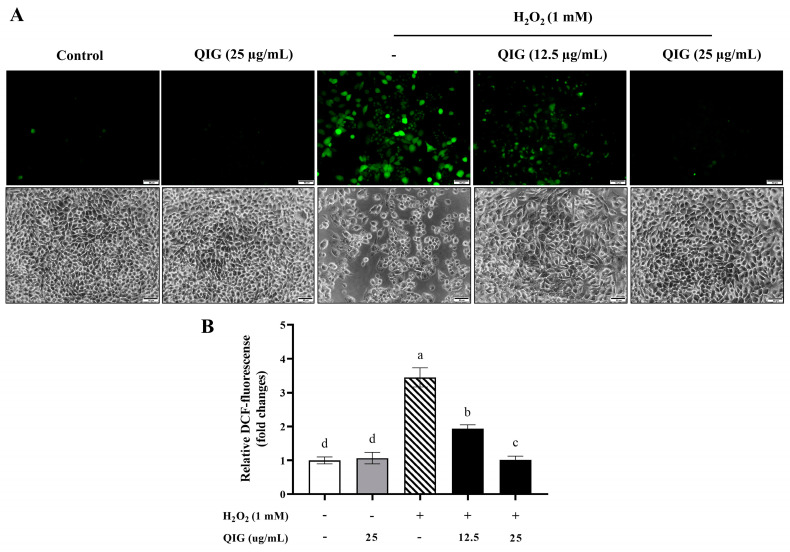
The effects of *Quercus infectoria* gall extract (QIG) on intracellular ROS generation upon H_2_O_2_-induced oxidative stress in L929 fibroblasts. Cells were treated with either 25 µg/mL QIG, 1 mM H_2_O_2_, or 1 mM H_2_O_2_ together with different doses of QIG (12.5 and 25 µg/mL) for 24 h. (**A**) Intracellular ROS levels were measured by 2′,7′-dichlorofluorescein diacetate (DCF-DA) staining. The intracellular ROS formation was observed under a fluorescent microscope at a magnification of 200×. Scale bar = 50 µm. (**B**) Relative fluorescence intensity results. The values are expressed in fold changes compared with the control group (untreated cells). Values are representative of three independent experiments performed in triplicate and are expressed as mean ± SD (error bars). Different letters indicate statistically significant differences (*p* < 0.05).

Oxidative stress is the result of an imbalance in the synthesis and accumulation of ROS in cells. Overproduction of ROS leads to oxidative stress, which is associated with various biological processes, including DNA damage, inflammation, and apoptosis [38]. A previous study demonstrated that H_2_O_2_ drastically increased intracellular ROS production and accumulation in fibroblasts [5]. Thus, to investigate the mechanism underlying the protective effects of *Q. infectoria* gall extract against H_2_O_2_-induced cytotoxicity, intracellular ROS formation was evaluated. L929 cells were treated with H_2_O_2_, *Q. infectoria* gall extract, or H_2_O_2_ together with *Q. infectoria* gall extract for 24 h. The amount of intracellular ROS was determined using a cell-permeant dye (DCFH-DA). Our results showed that treatment with H_2_O_2_, but not the extract, significantly increased the intracellular ROS production in L929 cells (Figure 4A). The relative fluorescence intensity was analyzed in comparison to the control group. The amount of intracellular ROS generation in H_2_O_2_-treated cells was approximately three times higher than the control (3.45 ± 0.28 vs. 1.00 ± 0.10, *p* < 0.05) (Figure 4B). This indicates that H_2_O_2_ induced oxidative stress in L929 cells. Notably, when *Q. infectoria* gall extract was introduced to the cells along with H_2_O_2_, the levels of intracellular ROS production significantly decreased in a dose-related manner compared to the treatment with H_2_O_2_ alone (1.93 ± 0.11 of 12.5 µg/mL QIG + H_2_O_2_ and 1.01 ± 0.11 of 25 µg/mL QIG + H_2_O_2_ vs. 3.45 ± 0.28 of H_2_O_2_ alone, *p* < 0.05). Furthermore, *Q. infectoria* gall extract at a concentration of 25 µg/mL completely inhibited H_2_O_2_-induced cellular ROS production, with ROS levels comparable to the control group (1.01 ± 0.11 vs. 1.00 ± 0.10, *p* > 0.05) (Figure 4A,B). These findings suggest that *Q. infectoria* gall extract protects L929 fibroblasts from H_2_O_2_-induced cytotoxicity by mitigating ROS formation and oxidative damage in cells.

### 3.6. Comparative Effects of Quercus infectoria Gall Extract and the Main Components Present in the Nutgall on H_2_O_2_-Induced Damage in L929 Cells

**Figure 5:**
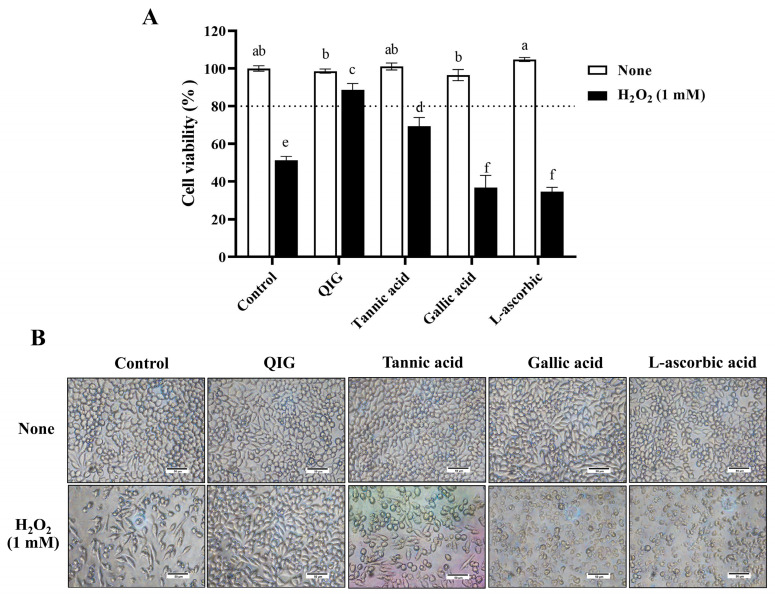
Comparative effects of *Q. infectoria* gall extract (QIG) and the main components presented in the nutgall on H_2_O_2_-induced injury in L929 fibroblasts. Cells were treated with 1 mM of H_2_O_2_ in the presence or absence of either QIG, tannic acid, gallic acid, or L-ascorbic acid at a concentration of 25 µg/mL for 24 h. (**A**) Cell viability was assessed using an MTT assay. The values are expressed as relative to the control group (untreated cells). Values are representative of three independent experiments performed in triplicate and are expressed as mean ± SD (error bars). Different letters indicate statistically significant differences (*p* < 0.05). (**B**) Representative microscopic images at a magnification of 200×. Scale bar = 50 µm.

To compare the effects of the pure compounds on H_2_O_2_-induced cytotoxicity in L929 fibroblasts, cells were treated with either 25 µg/mL of tannic acid, gallic acid, L-ascorbic acid, or *Q. infectoria* gall extract in the presence or absence of 1 mM H_2_O_2_. The results showed that none of these compounds exhibited cytotoxic effects on L929 cells at the tested concentration. Treatment with H_2_O_2_ remarkably decreased cell viability to 51.22 ± 2.12%. However, introducing tannic acid along with H_2_O_2_ significantly increased cell viability to 69.29 ± 4.67%, suggesting the protective role of tannic acid against H_2_O_2_-induced cytotoxicity. This is consistent with a previous study demonstrating that tannic acid treatment could improve cell viability, cell antioxidant enzymes, and stimulate the Nrf2 pathway against oxidative injury in IPEC-J2 cells [30]. Surprisingly, the combination of H_2_O_2_ with gallic acid or L-ascorbic acid resulted in higher cytotoxicity than treatment with H_2_O_2_ alone (cell viability was 36.88 ± 6.37% and 34.54 ± 2.39% vs. 51.22 ± 2.12%) (Figure 5A,B). This finding is consistent with a previous finding. The mixture of ascorbic acid and H_2_O_2_ significantly reduced the viability of murine neuroblastoma cells more than the treatment with H_2_O_2_ alone [39]. This phenomenon may be attributed to the pro-oxidant properties of ascorbic acid under certain conditions. For example, the reaction between ascorbic acid and DMEM culture medium has been shown to generate H_2_O_2_ [40]. Similarly, it was discovered that the incubation of gallic acid and phosphate buffer (pH 7.4) also increased H_2_O_2_ formation [41]. Consequently, combining L-ascorbic acid or gallic acid with H_2_O_2_ in a culture medium may result in a greater H_2_O_2_ concentration, thereby increasing cytotoxicity.

Compared to the antioxidant activity of gallic acid, the gall extract demonstrated lower antioxidant capacity (Table 2). However, it was more effective in protecting L929 cells against H_2_O_2_-induced cytotoxicity (cell viability was 36.88 ± 6.37% vs. 88.74 ± 3.24%, *p* < 0.05, Figure 5). These results indicate that the protective effect against H_2_O_2_-induced cytotoxicity is not solely dependent on antioxidant activity. The chemical composition, concentration, and ratio of the compounds should be considered. In addition, our data revealed that the extract from *Q. infectoria* gall exhibited superior efficacy compared with the pure compounds. This suggests that the synergistic interactions among the various bioactive constituents within the extract may play a crucial role in its enhanced biological activity, highlighting the potential benefits of utilizing whole extracts in therapeutic applications over individual isolated compounds.

### 3.7. Quercus infectoria Gall Extract Protects against H_2_O_2_-Induced Injury in L929 Fibroblasts at Various Time Intervals

**Figure 6:**
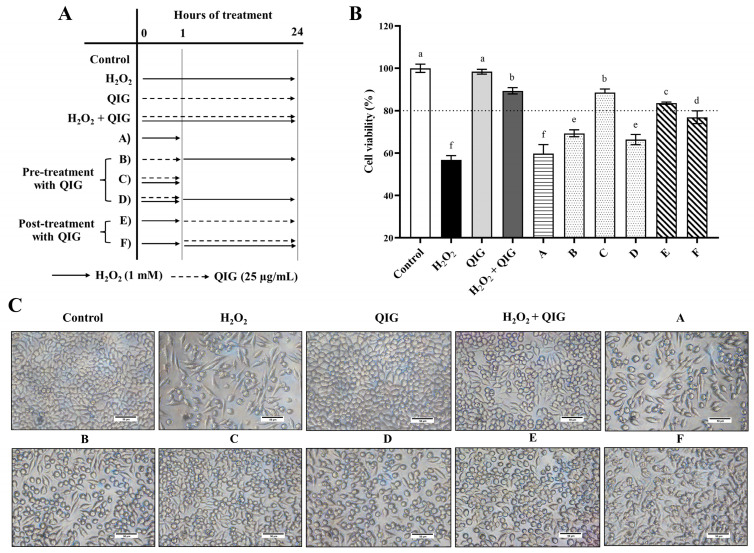
The protective effects of *Quercus infectoria* gall extract (QIG) against H_2_O_2_-induced injury in L929 fibroblasts at different time intervals. (**A**) Scheme showing the treatment of L929 fibroblasts. Cells were treated with 1 mM of H_2_O_2_ (solid line arrow) and/or 25 µg/mL of QIG (dashed arrow) at various time points. (**B**) Cell viability was assessed using an MTT assay. The values are expressed as relative to the control group (untreated cells). Values are representative of three independent experiments performed in triplicate and are expressed as mean ± SD (error bars). Different letters indicate statistically significant differences (*p* < 0.05). (**C**) Representative microscopic images at a magnification of 200×. Scale bar = 50 µm.

According to the results presented in Figure 3, *Q. infectoria* gall extract could protect against H_2_O_2_-induced cytotoxicity when cells were treated with the extract and H_2_O_2_ simultaneously. To further analyze the protective effects of the gall extract against H_2_O_2_-induced cytotoxicity, we treated L929 cells with 1 mM H_2_O_2_ and/or 25 µg/mL *Q. infectoria* gall extract at different time points, as shown in Figure 6A. The treatment was divided into two phases: (1) a short period of 1 h and (2) a long period of 23 h. First, we observed that the cell viability after exposure to H_2_O_2_ for 24 h or after exposure to H_2_O_2_ for 1 h followed by fresh culture medium for 23 h (Figure 6A, condition A) demonstrated no significant difference (56.74 ± 2.11% vs. 59.75 ± 4.27%, *p* > 0.05). It has been reported that H_2_O_2_ noticeably induced DNA damage in HepG2 cells within 1 h of treatment. However, the damage levels gradually decreased and returned to the normal stage within 24 h, suggesting cellular adaptation [42]. In addition, H_2_O_2_ can be degraded by cellular catalase [43]. Thus, the amount of H_2_O_2_ after 1 h may be insufficient to cause cytotoxicity. Taken together, these data indicate that the first hour of treatment is a critical period for H_2_O_2_-induced damage in cells.

As expected, *Q. infectoria* gall extract can protect against H_2_O_2_-induced injury at any stage of treatment. Co-treatment (H_2_O_2_ + QIG), pre-treatment (Figure 6A, conditions B–D), and post-treatment (Figure 6A, conditions E and F) with the extract significantly increased the cell viability of L929 fibroblasts compared to cells treated with H_2_O_2_ alone (*p* < 0.05) (Figure 6B,C). A prior study demonstrated that the gall extract effectively scavenges free radicals such as hydroxyl radicals (^•^OH) and H_2_O_2_, with IC_50_ values of 6 g/mL and 2.6 g/mL, respectively [44]. It can be hypothesized that when cells are treated simultaneously with *Q. infectoria* gall extract and H_2_O_2_, the extract may directly scavenge H_2_O_2_ before it exhibits cytotoxicity. Additionally, pre-treatment and post-treatment with the extract could also ameliorate H_2_O_2_-induced cytotoxicity, indicating that *Q. infectoria* gall extract may enhance cellular antioxidant responses in the fibroblasts. Our findings provide important evidence for the anti-H_2_O_2_ capabilities of *Q. infectoria* gall extract, demonstrating its ability to both prevent and restore H_2_O_2_-induced damage in L929 cells.

### 3.8. Quercus infectoria Gall Extract Inhibits Inflammation and Restores the Gene Encoding Type I Procollagen Chains in H_2_O_2_-Treated Fibroblasts

**Figure 7:**
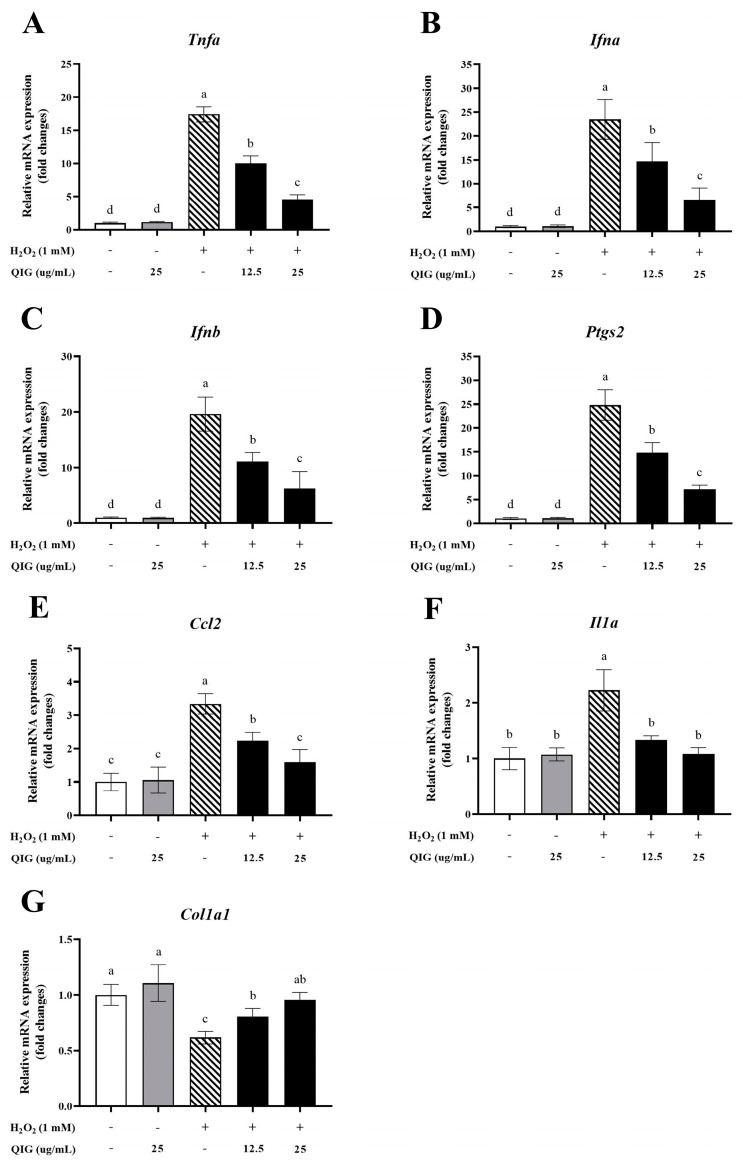
The effects of H_2_O_2_ and *Quercus infectoria* gall extract (QIG) on the expression of genes associated with inflammation and collagen synthesis in fibroblasts. Cells were treated with either 25 µg/mL QIG, 1 mM H_2_O_2_, or 1 mM H_2_O_2_ together with different doses of QIG (12.5 and 25 µg/mL) for 6 h. (**A**–**F**) RT-qPCR results for the expression of inflammation-related genes: *Tnfa*, *Ifna*, *Ifnb*, *Ccl2*, *Ptgs2*, and *Il1a*. (**G**) RT-qPCR analysis of the gene encoding collagen type 1 alpha 1 (*Col1a1*). Gene expression was normalized with *Actb*. The data are expressed in fold changes compared with the control group (untreated cells). Values are representative of three independent experiments performed in triplicate and are expressed as mean ± SD (error bars). Different letters indicate statistically significant differences (*p* < 0.05).

ROS are closely associated with inflammation. A recent study showed that fibroblasts, in addition to immune cells, also produce several pro-inflammatory genes upon exposure to inflammatory stimuli [45,46]. Although an inflammatory response is a key step in wound healing, chronic inflammation can delay the healing process [47]. Extensive inflammation can decrease fibroblast proliferation, impair cell migration, induce extracellular matrix degradation, and suppress collagen synthesis [48]. To assess whether *Q. infectoria* gall extract prevents H_2_O_2_-induced inflammation, the expression of inflammation-related genes was examined by RT-qPCR. As depicted in Figure 7A–F, treatment with H_2_O_2_ significantly increased the expression of *Tnfa*, *Ifna*, *Ifnb*, *Ccl2*, *Ptgs2*, and *Il1a* genes compared with the control group, indicating that H_2_O_2_ can trigger an inflammatory response in L929 fibroblasts. The gall extract alone did not alter the gene expression. However, combining the extract with H_2_O_2_ significantly reduced the expression levels of these inflammation-related genes in a dose-dependent manner (Figure 7A–F), suggesting that *Q. infectoria* gall extract potentially prevented H_2_O_2_-induced inflammation in cells.

Collagen, a major component of the extracellular matrix, is crucial for new tissue formation and wound repair. An excessive amount of ROS has been reported to degrade collagen and inhibit the transforming growth factor-β pathway, leading to a decrease in collagen production [10]. Thus, to examine whether *Q. infectoria* gall extract is involved in collagen synthesis in fibroblasts, the mRNA levels of collagen type I alpha 1 chain (*Col1a1*) were evaluated. The results in Figure 7G showed that the expression of the *Col1a1* gene in L929 cells significantly decreased upon exposure to H_2_O_2_ (0.62 ± 0.06 relative fold-change compared to the untreated control, *p* < 0.05). Notably, the extract alone did not increase the mRNA levels of *Col1a1*, but it can prevent H_2_O_2_-induced reduction in the *Col1a1* gene in a dose-dependent manner (relative fold-change to the untreated control was 0.81 ± 0.07 of 12.5 µg/mL QIG + H_2_O_2_ and 0.95 ± 0.07 of 25 µg/mL QIG + H_2_O_2_ vs. 0.62 ± 0.06 of H_2_O_2_ alone, *p* < 0.05). These findings suggest that *Q. infectoria* gall extract not only counteracts H_2_O_2_-induced inflammation but also protects against the reduction in collagen synthesis caused by oxidative stress.

### 3.9. H_2_O_2_-Induced Delays in Wound Healing of L929 Fibroblasts

**Figure 8:**
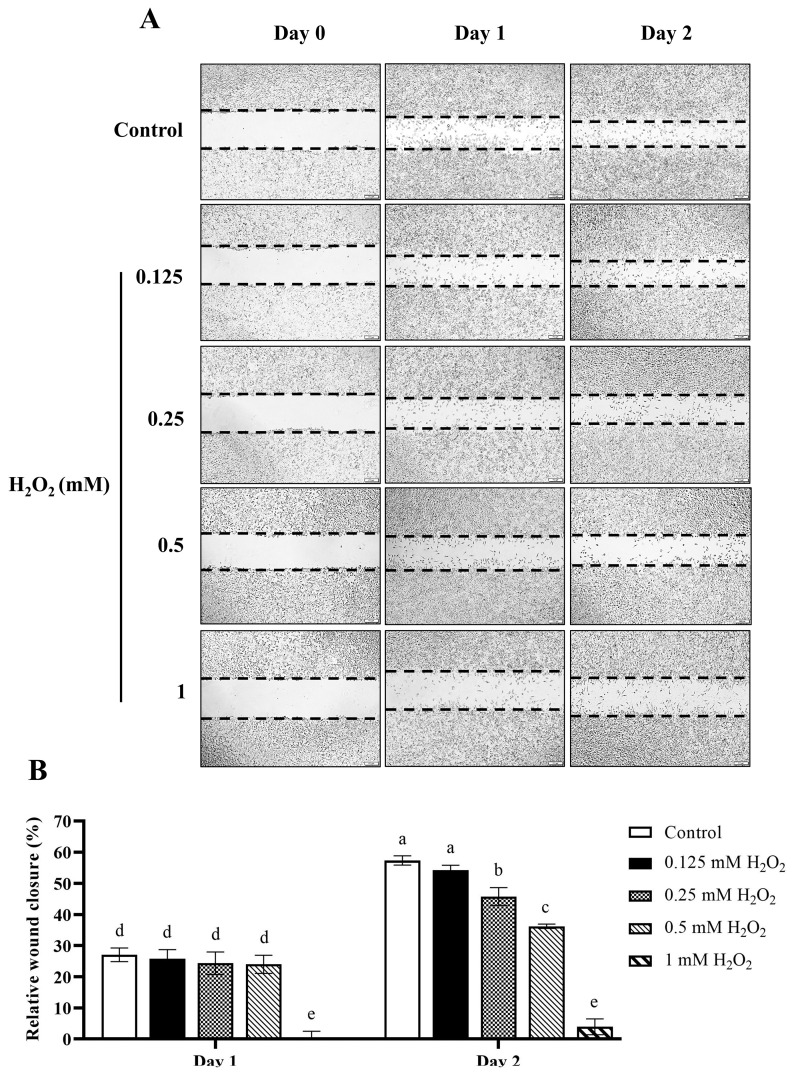
The effects of H_2_O_2_ on L929 fibroblasts wound healing. (**A**) A scratch assay of L929 cells treated with various concentrations of H_2_O_2_ (0.125 to 1 mM)**.** The images represent cell migration at 0, 24, and 48 h of treatment. The area of the wound is defined by the dashed lines. (**B**) Quantification of wound closure rates (%), defined as the difference in wound area from day 0 of each group. Values are representative of three independent experiments performed in triplicate and are expressed as mean ± SD (error bars). Different letters indicate statistically significant differences (*p* < 0.05).

The scratch wound assay was conducted to investigate the wound healing of fibroblasts. First, we examined the effects of H_2_O_2_ on L929 cell migration. After wounding, cells were treated with various concentrations of H_2_O_2_ ranging from 0.125 to 1 mM. The wound closure rate was calculated and expressed as the percentage relative to the wound at time zero (considered 0% wound closure). The data revealed a clear dose-dependent inhibition of wound closure following H_2_O_2_ treatment. At higher concentrations, particularly at 1 mM, H_2_O_2_ almost completely halted the wound healing process compared with the control group (wound closure: 3.65 ± 5.99% vs. 27.07 ± 2.17% at day 1 and 4.15 ± 2.76% vs. 57.36 ± 1.46% at day 2, *p* < 0.05) (Figure 8). These findings strongly suggest that elevated oxidative stress, as induced by H_2_O_2_, significantly impairs fibroblast function, leading to delayed or inhibited wound healing. This finding is consistent with a previous in vivo study in which high levels of oxidative stress were shown to decrease the quality of healing, leading to the development of chronic wounds in diabetic mice [49]. Diabetic wounds are often characterized by an inability to heal properly, a condition closely linked to the presence of elevated ROS levels, which disrupt normal cellular functions. The observed delay in wound closure could be attributed to the detrimental effects of oxidative stress on various cellular processes critical for wound healing. Oxidative stress can cause significant damage to cellular components, including lipids, proteins, and DNA, which are essential for cell migration, proliferation, and tissue repair [6]. Given these findings, it is evident that managing oxidative stress levels is crucial in therapeutic strategies aimed at enhancing wound healing. Antioxidants and other agents that can mitigate oxidative stress might offer significant benefits in improving healing outcomes, especially in cases where chronic wounds or high oxidative stress conditions are present.

### 3.10. Quercus infectoria Gall Extract Facilitates Fibroblast Wound Healing under Conditions of H_2_O_2_-Induced Oxidative Stress

**Figure 9:**
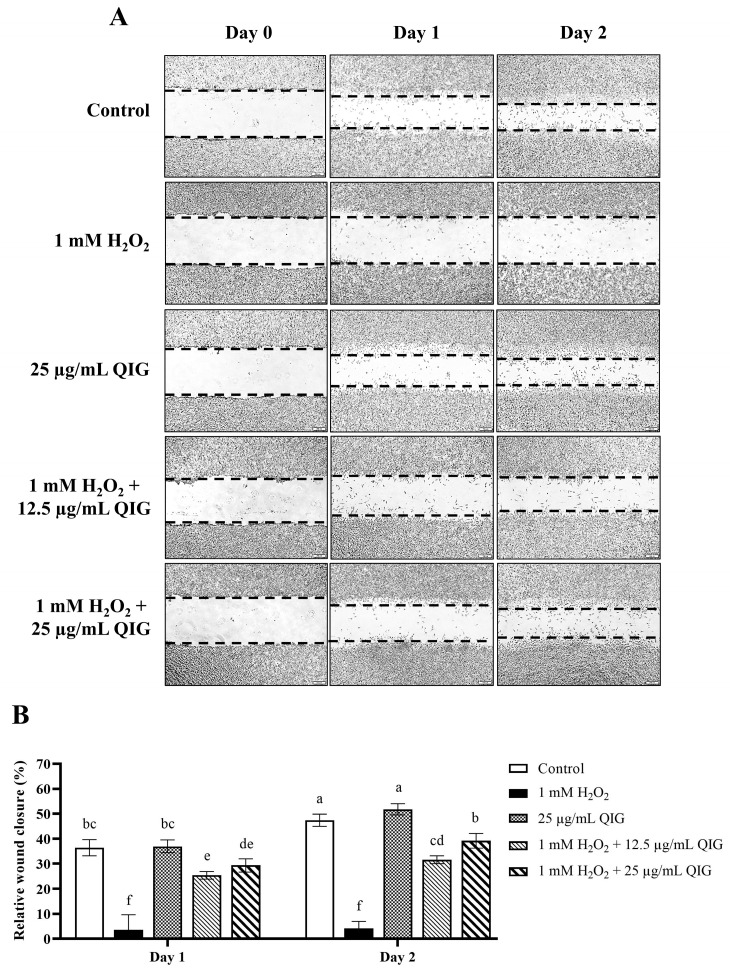
The wound healing effects of *Quercus infectoria* gall extract (QIG) on L929 fibroblasts under H_2_O_2_-induced oxidative stress. (**A**) A scratch assay of L929 cells treated with either 25 µg/mL QIG, 1 mM H_2_O_2_, or 1 mM H_2_O_2_ together with different doses of QIG (12.5 and 25 µg/mL). The images represent cell migration at 0, 24, and 48 h of treatment. The area of the wound is defined by the dashed lines. (**B**) Quantification of wound closure rates (%), defined as the difference in wound area from day 0 of each group. Values are representative of three independent experiments performed in triplicate and are expressed as mean ± SD (error bars). Different letters indicate statistically significant differences (*p* < 0.05).

To examine the effect of *Q. infectoria* gall extract on the wound healing of fibroblasts under oxidative stress, we performed the assay similarly to the method above, except that the extract was introduced to the cells in the presence or absence of 1 mM H_2_O_2_. As shown in Figure 9, the area of wound closure between the gall extract treatment and the control group showed no significant difference (wound closure: 36.97 ± 2.55% vs. 36.43 ± 3.19% at day 1, and 51.78 ± 2.23% vs. 47.39 ± 2.47% at day 2, *p* > 0.05), indicating that the extract at the concentration used in this study did not enhance the wound healing rate in fibroblasts. Interestingly, combining the extract with H_2_O_2_ significantly promoted wound closure in a dose- and time-dependent manner more than treatment with H_2_O_2_ alone (wound closure at day 1: 25.38 ± 1.52% of 12.5 µg/mL QIG + H_2_O_2_ and 29.36 ± 2.65% of 25 µg/mL QIG + H_2_O_2_ vs. 3.65 ± 5.99% of H_2_O_2_ alone, *p* < 0.05) (wound closure at day 2: 31.63 ± 1.63% of 12.5 µg/mL QIG + H_2_O_2_ and 39.22 ± 2.96% of 25 µg/mL QIG + H_2_O_2_ vs. 4.15 ± 2.76% of H_2_O_2_ alone, *p* < 0.05).

Previous studies revealed that streptozotocin induced oxidative stress both in vitro and in vivo, contributing to the development of diabetic complications and impaired wound healing [50,51,52]. In this context, both *Q. infectoria* formulations and *Q. infectoria* gall hydroethanolic extract were shown to promote wound healing in streptozotocin-induced diabetic animals [20,21]. The findings from our current study further suggest that *Q. infectoria* gall extract may play a critical role in alleviating streptozotocin-induced oxidative stress. By reducing oxidative stress, the extract appears to diminish inflammation in the wound area, which is a key barrier to effective healing. This reduction in inflammation can facilitate increased fibroblast migration and collagen deposition, thereby improving the overall rate of wound healing. These insights underscore the potential of *Q. infectoria* gall extract as a promising therapeutic agent, particularly for enhancing wound healing in conditions associated with oxidative stress, such as diabetic wounds.

## 4. Conclusions

This study demonstrated that H_2_O_2_ significantly induces cytotoxicity, oxidative stress, and inflammation in L929 skin fibroblasts, leading to impaired wound healing. To the best of our knowledge, this is the first study illustrating that the ethanolic extract of *Q. infectoria* galls effectively mitigates these adverse effects by protecting against H_2_O_2_-induced cytotoxicity, reducing intracellular ROS levels, and downregulating the expression of inflammation-related genes. Moreover, the extract significantly promotes wound closure in fibroblasts under oxidative stress conditions, highlighting its potential as a therapeutic agent for chronic wounds and oxidative stress-related diseases. While these results are promising, further study on the cellular antioxidant response is needed to fully understand the mechanisms by which *Q. infectoria* gall protects against oxidative stress.

## Figures and Tables

**Table 1:** Primer sequences for RT-qPCR used in this study.

Target Genes	Types	Primer Sequences 5′ to 3′
m*Tnfa*	Forward	CAGAAAGCATGATCCGCGAC
	Reverse	CGATCACCCCGAAGTTCAGT
m*Ifna*	Forward	GGATGTGACCTTCCTCAGACTC
	Reverse	ACCTTCTCCTGCGGGAATCCAA
m*Ifnb*	Forward	CCAGCTCCAAGAAAGGACGA
	Reverse	CGCCCTGTAGGTGAGGTTGAT
m*Ccl2*	Forward	GGCTCAGCCAGATGCAGTTAA
	Reverse	CCAGCCTACTCATTGGGATCA
m*Ptgs2*	Forward	AGCCCATTGAACCTGGACTG
	Reverse	ACCCAATCAGCGTTTCTCGT
m*Il1a*	Forward	ACGGCTGAGTTTCAGTGAGACC
	Reverse	CACTCTGGTAGGTGTAAGGTGC
m*Col1a1*	Forward	CCTCAGGGTATTGCTGGACAAC
	Reverse	CAGAAGGACCTTGTTTGCCAGG
m*Actb*	Forward	CATTGCTGACAGGATGCAGAAGG
	Reverse	TGCTGGAAGGTGGACAGTGAGG

**Table 2:** Antioxidant activity of *Q. infectoria* gall extract and the main components presented in the nutgall.

Samples	Total Antioxidant Capacity		
	(µM TE/µg Sample)		
	DPPH	ABTS	FRAP
*Q. infectoria* gall extract	305.43 ± 7.48 ^c^	508.94 ± 15.12 ^b^	442.08 ± 9.41 ^b^
Tannic acid	393.82 ± 8.10 ^b^	551.65 ± 14.03 ^b^	348.64 ± 20.21 ^c^
Gallic acid	745.24 ± 31.90 ^a^	1062.81 ± 41.68 ^a^	1101.90 ± 48.13 ^a^
L-ascorbic acid	153.02 ± 11.81 ^d^	223.70 ± 18.78 ^c^	131.73 ± 22.98 ^d^

Results are representative of three independent experiments performed in triplicate and are expressed as mean ± SD (error bars). All data were analyzed using ANOVA analysis followed by a post hoc Duncan test. Different letters indicate statistically significant differences (*p* < 0.05). TE: Trolox equivalents.

## Data Availability

The original contributions presented in the study are included in the article; further inquiries can be directed to the corresponding author.
